# Durable response of lung carcinoma patients to EGFR tyrosine kinase inhibitors is determined by germline polymorphisms in some immune-related genes

**DOI:** 10.1186/s12943-023-01829-4

**Published:** 2023-07-29

**Authors:** Lorraine Dalens, Julie Niogret, Corentin Richard, Sandy Chevrier, Pascal Foucher, Bruno Coudert, Aurélie Lagrange, Laure Favier, Virginie Westeel, Stefano Kim, Olivier Adotevi, Caroline Chapusot, Laurent Martin, Laurent Arnould, Courèche-Guillaume Kaderbhai, Romain Boidot

**Affiliations:** 1 Medical Oncology Department, Georges-François Leclerc Cancer Center – UNICANCER, Dijon, France; 2 Molecular Biology Unit, Department of Biology and Pathology of Tumors, Georges-François Leclerc Cancer Center – UNICANCER, Dijon, France; 3 Department of Thoracic Oncology, Dijon University Hospital, Dijon, France; 4 Chest Disease Department, University Hospital of Besançon, Besançon, France; 5 Department of Pathology, Dijon University Hospital, Dijon, France; 6 Pathology Unit, Department of Biology and Pathology of Tumors, Georges-François Leclerc Cancer Center – UNICANCER, Dijon, France; 7 UMR CNRS 6302, Dijon, France

**Keywords:** Lung carcinoma, Exome analysis, SNPs, Innate immunity, EGFR TKI

## Abstract

**Background:**

Non-small cell lung cancer is a very poor prognosis disease. Molecular analyses have highlighted several genetic alterations which may be targeted by specific therapies. In clinical practice, progression-free survival on EGFR TKI treatment is between 12 and 14 months. However, some patients progress rapidly in less than 6 months, while others remain free of progression for 16 months or even longer during EGFR TKI treatment.

**Methods:**

We sequenced tumor exomes from 135 lung cancer patients (79 with *EGFR*-wildtype (WT), 56 with *EGFR*-mutant tumors) enrolled in the ALCAPONE trial (genomic analysis of lung cancers by next generation sequencing for personalized treatment).

**Results:**

Some germline polymorphisms were enriched in the *EGFR*-mutant subset compared to *EGFR*-WT tumors or to a reference population. However, the most interesting observation was the negative impact of some germline SNPs in immunity-related genes on survival on EGFR TKI treatment. Indeed, the presence of one of three particular SNPs in the *HLA-DRB5* gene was associated with a decreased PFS on EGFR TKI*.* Moreover, some SNPs in the *KIR3DL1* and *KIR3DL2* genes were linked to a decrease in both progression-free and overall survival of patients with *EGFR*-mutant tumors.

**Conclusion:**

Our data suggest that SNPs in genes expressed by immune cells may influence the response to targeted treatments, such as EGFR TKIs. This indicates that the impact of these cells may not be limited to modulating the response to immunotherapies. Further studies are needed to determine the exact mechanisms underlying this influence and to identify the associated predictive and prognostic markers that would allow to refine treatments and so improve lung cancer patient outcomes.

**Trial registration:**

NCT02281214: NGS Genome Analysis in Personalization of Lung Cancer Treatment (ALCAPONE).

**Supplementary Information:**

The online version contains supplementary material available at 10.1186/s12943-023-01829-4.

## Introduction

Lung cancer is one of the most frequent cancers and one of the leading causes of cancer death worldwide. Extensive studies of the mutational landscape in lung carcinoma, particularly in non-small cell lung carcinoma (NSCLC), have revealed the existence of specific alterations, many of which have emerged as therapeutic targets or biomarkers predicting response to treatment over the past decade. The most well-known targetable alterations in NSCLC are mutations in the *EGFR* gene, *ALK* fusions, *ROS1* fusions, mutations in splicing sites around exon 14 of the *MET* gene, and—more recently added to the list of actionable variants—the p.(Val600Glu) mutation in the *BRAF* gene and the p.(Gly12Cys) mutation in the *KRAS* gene. Alterations in other genes, such as *KEAP1*, *STK11*, or *TP53,* have been described as biomarkers of response to immunotherapy.

Among therapeutics targeting these specific molecular alterations, EGFR tyrosine kinase inhibitors (TKIs) have been in use for nearly 20 years. While third generation EGFR TKIs, like osimertinib, have been approved as firstline treatment for metastatic NSCLC, first- and second-generation EGFR TKIs are still in use. Despite the overall efficacy of these targeted therapies in patients with *EGFR*-mutant tumors, some patients do not achieve a durable response to EGFR TKI treatment. Some mechanisms of resistance to EGFR TKIs have been described, including the emergence of the p.(Thr790Met) mutation in the *EGFR* gene, conferring resistance to the first- and second-generation inhibitors, as well as the *EGFR* p.(Cys797Ser) mutation, which confers resistance to the third-generation TKIs. However, reliable markers of durable treatment response remain elusive. To date, only two predictive markers have been described in the literature: sensitizing mutations in the *EGFR* gene and *CDK4/6* amplification, neither of which indicates a marker of durable treatment response.

Single Nucleotide Polymorphisms (SNPs) are common genetic variations found in all individuals. While most SNPs are neutral and do not affect protein function, some can influence protein activity. For example, SNPs in the *DPYD* gene have been linked to toxicity to 5-fluorouracil. Genome-wide studies have identified numerous SNPs that appear to be linked to susceptibility to different diseases or disorders.

In this study, we carried out whole-exome sequencing on 135 lung carcinomas with known *EGFR* mutational status (79 tumors with wild-type *EGFR* and 56 with *EGFR*-mutant tumors) to determine whether some variants may impact the durability of response to treatment in patients with *EGFR*-mutant tumors.

## Methods

### Study design and patients

The patients included in this study were participants in a multicenter, prospective clinical trial, ALCAPONE (NCT02281214) (genomic analysis of lung cancer by next-generation sequencing for personalization of treatment). This trial was initiated in November 2014, and aimed to investigate possible associations between NGS-identified genomic biomarkers and the effectiveness of some treatments in patients with advanced lung cancer. The participants were recruited from among patients treated at the Dijon Cancer Center (*Centre Georges-Francois Leclerc*), the Dijon University Hospital, or the Besançon University Hospital (France). A total of 165 patients were recruited between November 2014 and August 2018. Patients were considered as eligible if they presented a locally advanced inoperable or metastatic lung cancer. We only included patients with incurable disease. Detailed inclusion and exclusion criteria are available on ClinicalTrials.gov. The clinical data of the population was presented as Supplementary Results.

All patients signed an informed consent to participate in the trial. The trial protocol was approved by an institutional review committee and by the relevant French regional ethical committee (*Comité de protection des personnes Est*). Of note, the information on race/ethnicity of the study participants was not collected due to legal constraints (collecting such data is forbidden in France). However, based on the location of participating hospitals and the patients’ family names, we can presume that a vast majority of the patients were of Caucasian origins. Therefore, we used the Non-Finnish European (NFE) population (corresponding to Caucasian population) as a reference. In genetics, SNP frequency depends on ethnic origin, and some SNPs are specific to certain races. Hence, using a reference population that corresponds to the studied population is necessary.

### DNA isolation

DNA was isolated from formalin-fixed paraffin-embedded (FFPE) tumor tissue using the Maxwell 16 FFPE Plus LEV DNA Purification kit (Promega, Madison, WI, USA) following the manufacturer’s instructions. The tumor cell content was assessed by a pathologist on a hematoxylin-and-eosin-stained slide from the same core biopsy which was used for DNA extraction. Only samples containing more than 20% of tumor cells were included in the molecular analyses. The quantity of the extracted genomic DNA was assessed with a fluorimetric method using a Qubit device (Fisher Scientific, Waltham, MA, USA).

### Whole-exome capture and sequencing

The library was prepared from 200ng genomic DNA using the Agilent SureSelect XT reagent kit (AgilentTechnologies, SantaClara, USA) according to the manufacturer’s protocol. The totality of the enriched library was hybridized and captured with the SureSelect All Exon v5 (Agilent Technologies) baits. Following hybridization, the captured libraries were purified according to the manufacturer's recommendations and amplified by polymerase chain reaction (12 cycles). Normalized libraries were pooled and DNA was sequenced on an Illumina NextSeq500 device using 2*151 bp paired-end reads, and then multiplexed. Tumor DNA sequencing generated mean target coverage of 80X.

### Exome analysis workflow

Raw DNA sequencing data were aligned to the hg19 genome built using the Burrows-Wheeler Aligner (BWA), version 0.7.15. Duplicates were marked with Picard version 2.5.0. Base quality scores recalibration and variant calling were performed using GATK tools, version 3.6. Single Nucleotide Variations were annotated using the Variant Studio Illumina software. Filters of candidate variants included the coverage depth of 10X or higher and a variant nucleotide allelic fraction in tumor DNA above 5%.

All genomic analyses were performed at the Dijon Cancer Center.

### Statistical analysis

To decrease the number of nucleotide alterations to test and decrease the probability of false-positive results, we have applied filters to select variants (SNPs and somatic mutations) present in more than 10% of individuals but in less than 90% of patients. These filters were applied on all groups or subsets on which we performed statistical analyses (full cohort, mutated EGFR patients, WT EGFR patients, PFS, OS…) and only the common variants were kept for the analysis. This step enabled us to select only 461 SNPs for statistical analysis.

Differences between the two groups were assessed using the Chi-Square test for categorial variables and the Student’s t-test for continuous variables. The objective response rate (ORR) was defined as the proportion of patients with a complete response or partial response to treatment according to the Response Evaluation Criteria in Solid Tumors (RECIST). Progression-free survival (PFS) was calculated as the time from the first metastatic treatment initiation to either evidence of progression, or death. Overall survival (OS) was defined as the time from the first metastatic treatment initiation to death. Both survival parameters were censored at the last follow-up timepoint if no event was recorded. The Kaplan–Meier estimator was used to calculate survival proportions. Log-rank tests were performed to assess the difference in survival between the groups. Univariate analyses aiming to determine the possible association of different DNA variants with PFS and OS were performed using Cox proportional hazard regression models in order to calculate hazard ratios (HR) with 95% confidence intervals. All *p-values* were adjusted for multi-testing by using the Benjamini–Hochberg procedure. All statistical tests were two-sided with *p*-values below 0.01 considered as indicative of a statistical significance. Statistical analyses were performed with R language v4.1.0 (R Core Team, 2021) and GraphPad Prism software v8.0.2 (GraphPad Software, La Jolla, CA, USA).

## Results

### Some germline polymorphisms are enriched in EGFR-mutant tumors

It is well known that *EGFR* mutations are mutually exclusive with some other gene variants in lung cancers, especially those in the *KRAS* gene. The analysis of all variants detected with whole-exome sequencing confirmed that only *EGFR*-WT tumors had *KRAS* mutations, with 17 tumors (21.52%) harboring gain-of-function mutations in this gene. Interestingly, three other genes were also altered only in *EGFR*-WT tumors: *KEAP1 (*20 patients; 25.32%), *STK11* (16 patients; 20.25%), and *UNC80* (11 patients; 13.92%; Supplementary Figure S3A). In the subset of *EGFR*-mutant tumors, all variants found in these three genes were Single Nucleotide Polymorphisms (SNPs; Supplementary Table S2). These gene variants were present in 43 (54.43%) patients with *EGFR*-WT tumors. Twenty-seven (34.18%) of them had variants in one gene, 11 (13.92%) had tumor variants in two genes, and 5 (6.33%) in all the three genes (Supplementary Figure S3A).

In contrast, we did not observe any specific variants occurring only in *EGFR*-mutant tumors. Nevertheless, some variants – classified as SNPs - were enriched in *EGFR*-mutant tumors compared to *EGFR*-WT tumors (Supplementary Fig. 3B, Supplementary Table S3). The *CHRNA3* c.67_69del variant was significantly more frequent in *EGFR*-mutant tumors compared to *EGFR*-WT tumors (*p* < 0.001). The same association was observed for three other SNPs: *COL18A1* c.3378_3386del (*p* = 0.001), *PLBD1* c.74_76del (*p* < 0.001), and *ZFPM1* c.1335_1340del (*p* < 0.001).

### Polymorphisms in genes of the antigen processing and presentation pathway are linked to worse PFS on EGFR-TKI

**Fig. 1:**
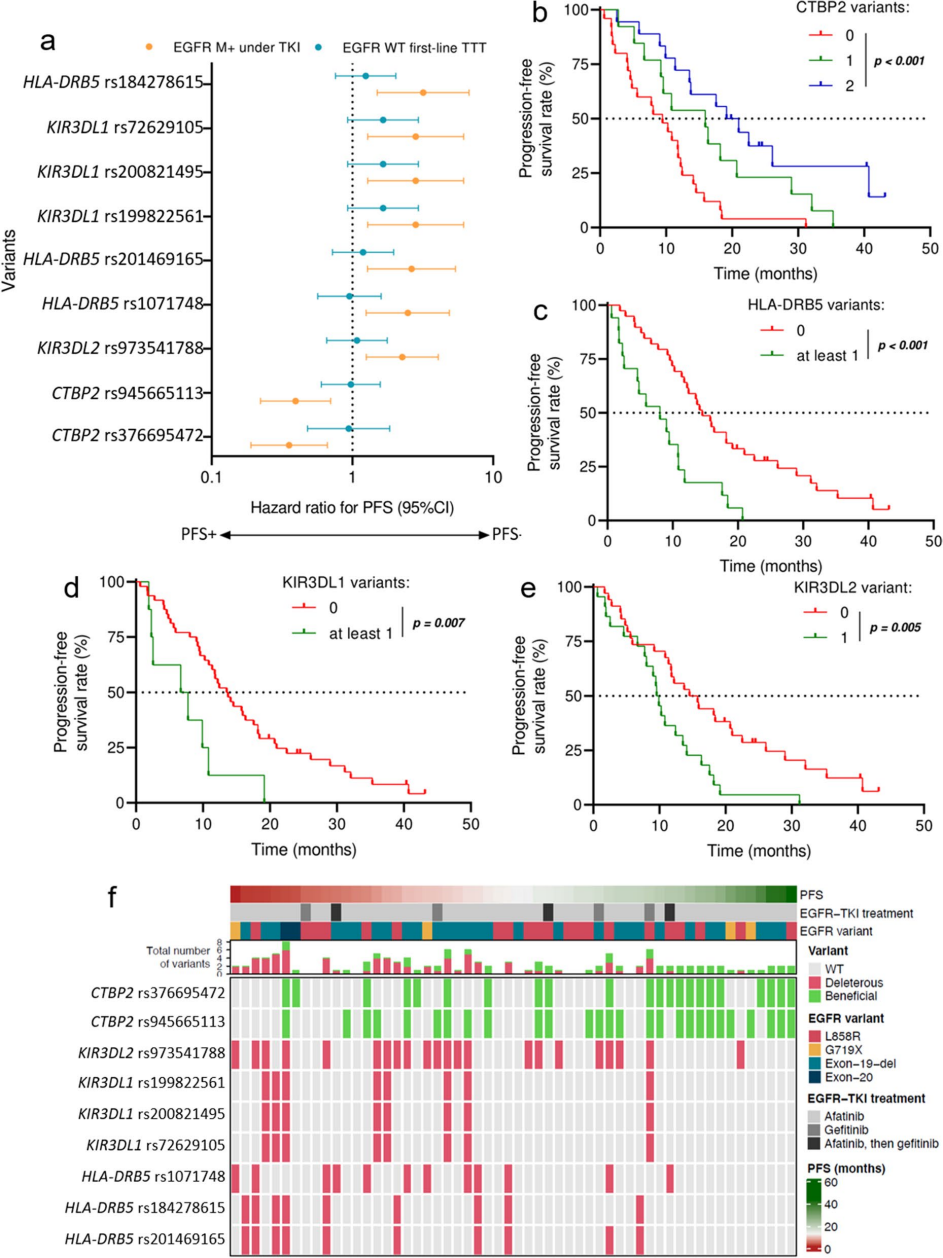
The impact of genetic variants on progression-free survival (PFS) of lung carcinoma patients with *EGFR*-mutant (M +) or *EGFR*-wildtype (WT) tumors. **A** Forest plot showing the effects of variants with a significant impact on PFS of patients with *EGFR* M + patients treated with EGFR tyrosine kinase inhibitors (TKIs) compared to PFS of patients with *EGFR*-WT tumors receiving another firstline treatment. **B-E** Kaplan–Meier curves for PFS. Patients with *EGFR*-mutant tumors were stratified according to the number of variants in the *CTBP2* gene (**B**), the absence or presence of at least one variant in the *HLA-DRB5* (**C**), in *KIR3DL1* (**D**), or in the *KIR3DL2* gene (**E**). **F** Heatmap summarizing the effects of all these variants on PFS of patients with *EGFR* M + tumors

We then analyzed whether some genetic variants might impact objective response to first-line treatment and progression-free survival of patients. We confirmed that mutations in exon 20 of the *EGFR* gene were associated with lack of response to treatment and negatively affected PFS (Supplementary Figure S1A). Hence, we excluded the two patients with exon-20 mutations from further analyses. No variant was significantly associated with the rate of objective response to treatment. However, polymorphisms in five genes impacted PFS of patients receiving EGFR TKIs (Fig. 1A). Two SNPs were found in the *CTBP2* gene: rs376695472 (c.2036 T > C, p.(Ile679Thr)) and rs945665113 (c.2059C > T, p.(Arg687Trp)), and both of them were associated with a better PFS of patients receiving EGFR TKI treatment (HR = 0.36 [0.19–0.67]; *p* = 0.0009 and HR = 0.39 [0.22–0.70]; *p* = 0.0012). Conversely, these SNPs did not affect PFS of patients with *EGFR*-WT tumors (Fig. 1A). The presence of both *CTBP2* variants in the tumor was associated with a median PFS of 20.1 months, the presence of either of the two SNPs was associated with a median PFS of 15.9 months, while the patients lacking both variants had a median PFS of 9.5 months (Fig. 1B).

Seven polymorphisms spread along three other genes: *HLA-DRB5, KIR3DL1,* and *KIR3DL2,* were associated with a worse PFS in patients receiving an EGFR TKI treatment. These included the following three variants in the *HLA-DRB5 gene*: rs1071748 (c.119A > G, p.(Asp40Gly)), rs184278615 (c.160C > T, p.(Arg54Trp)), and rs201469165 (c.163 T > A, p.(Phe55Ile)) (HR = 2.52 [1.24–5.09] *p* = 0.008, HR = 3.49 [1.58–7.68] *p* = 0.001, and HR = 2.78 [1.31–5.89] *p* = 0.005, respectively (Fig. 1A)). Similar to the two polymorphisms in the *CTBP2 gene*, the negative effect on PFS was observed only for patients with *EGFR*-mutant tumors. Overall, patients with tumors that did not harbor any of these three variants had better PFS than those with at least one of them (median PFS 14.6 versus 8.1 months; *p* < 0.001; Fig. 1C). Similarly, three polymorphisms in the *KIR3DL1* gene: rs199822561 (c.1277 T > C, p.(Ile426Thr)), rs200821495 (c.1279 T > A, p.(Leu427Met)), and rs72629105 (c.1286C > T, p.(Thr429Met)), were associated with a decreased PFS only in patients with *EGFR*-mutant tumors treated with an EGFR TKI (HR = 2.79 [1.26–6.14]; *p* = 0.008; Fig. 1A). The presence of at least one of these three polymorphisms in the tumor decreased the median PFS from 13.6 months to 7.2 months (Fig. 1D). A similar effect was found for the rs973541788 (c.1156C > G, p.(Gln386Glu)) polymorphism in the *KIR3DL2* gene: worse PFS for patients with mutant tumors compared to that of patients whose tumors did not harbor this variant (HR = 2.32 [1.27–4.24] *p* = 0.005) (Fig. 1A). The presence of the SNP decreased the median PFS from 15.2 to 9.7 months (Fig. 1E). The negative impact of all variants except those in the *CTBP2* gene on PFS, as well as the positive effect of the latter were consistent with the heatmap representation (Fig. 1F). Another SNP—in the *MUC17* gene—was also linked to worse PFS, but this effect was not specific to patients with *EGFR*-mutant tumors.

### Some polymorphisms in immunity-related genes are linked to better OS of patients receiving EGFR-TKI

**Fig. 2:**
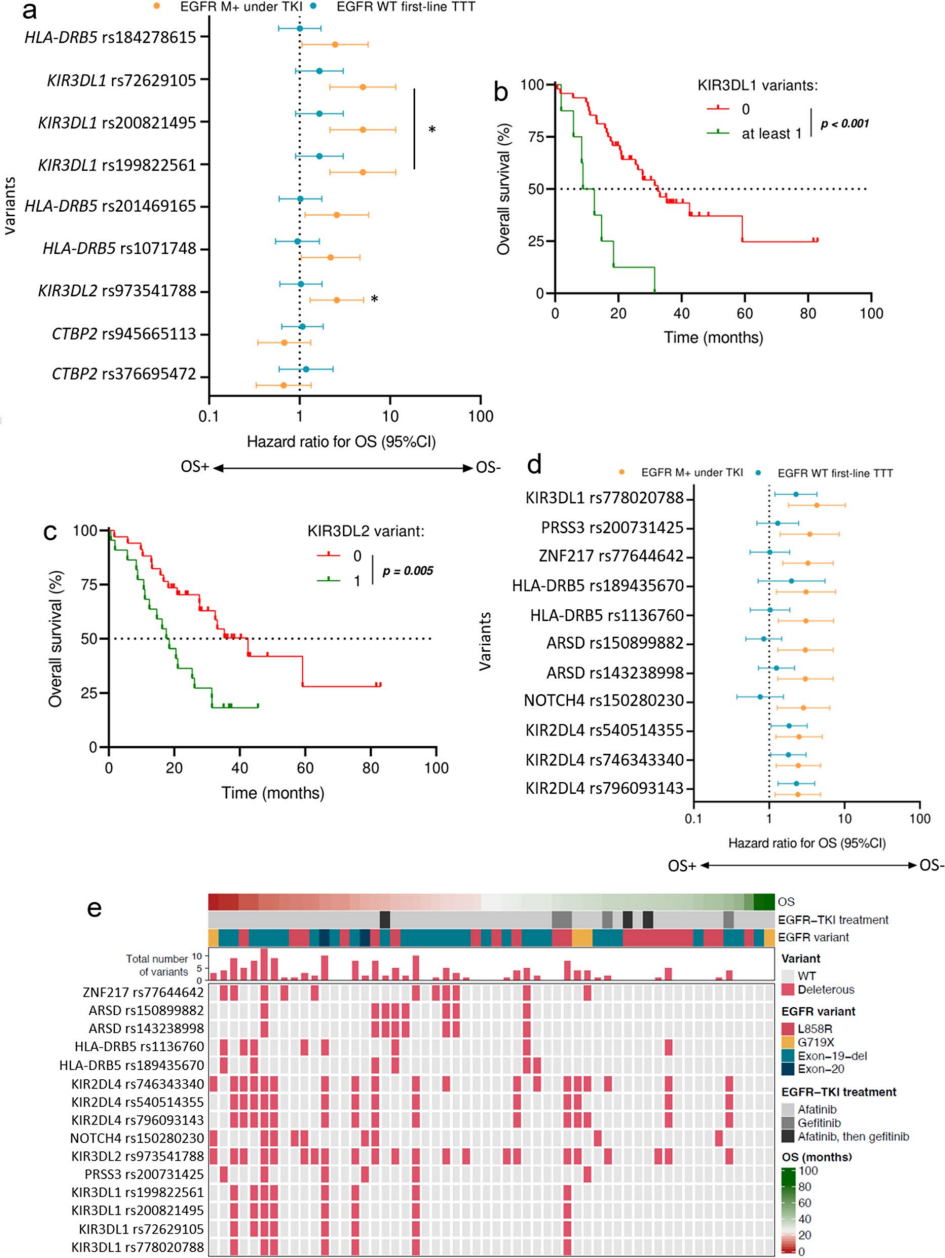
Genetic variants and overall survival (OS) of lung carcinoma patients with *EGFR*-mutant (M +) or *EGFR*-wildtype (WT) tumors. **A** Forest plot showing how variants with a significant impact on PFS affect the OS of patients with *EGFR* M + tumors treated with EGFR TKIs (in orange). Effects on the OS of patients with *EGFR*-WT tumors are displayed in blue. **B**, **C** Kaplan–Meier curves for PFS. Patients with *EGFR*-mutant tumors were stratified according to (**B**) the absence or presence of at least one variant in the *KIR3DL1* gene, or (**C**) the absence or presence of the variant in the *KIR3DL2* gene. **D** Forest plot illustrating the effect of variants with a significant impact on OS for patients with *EGFR* M + tumors treated with EGFR TKIs (in orange). The effects on the OS of patients with *EGFR*-WT tumors are displayed in blue. **E** Heatmap summarizing the effect of all these variants on the OS of patients with *EGFR* M + tumors

Next, we wondered whether the positive effect of different SNPs on PFS of patients receiving an EGFR TKI treatment affected their OS as well. We found that among the nine SNPs impacting PFS, only those in the *KIR3DL1* and *KIR3DL2* genes were significantly associated with a worse OS (Fig. 2A). Indeed, the presence of at least one of the following *KIR3DL1* SNPs: rs72629105, rs200821495, or rs199822561, was associated with a worse OS in patients with *EGFR*-mutant tumors (median OS of 10.6 months versus 32.5 months; *p* < 0.001; Fig. 2B).

The same association was observed for the rs973541788 variant in the *KIR3DL2* gene, with a median patient OS of 18 months if the variant was present in the tumor versus 42.6 months in case of its absence (Fig. 2C). Moreover, we tested whether other variants detected by whole-exome sequencing might influence OS of patients with *EGFR*-mutant tumors. In addition to the four above-mentioned SNPs impacting both PFS and OS, we found that eleven other variants, mainly in genes encoding proteins involved in antigen processing and presentation, were linked to a worse OS only in the subset of patients with *EGFR*-mutant tumors (Fig. 2D, Supplementary Table S4). Indeed, it appeared that the frequency of these variants was higher in patients with a poor OS (Fig. 2E).

## Discussion

The use of targeted therapies in lung cancer has revolutionized patient care in the past several years, especially for patients with *EGFR*-mutant tumors treated with EGFR TKIs. However, despite the specificity of EGFR TKIs, some patients do not experience long-lasting benefits from these treatments. Even with the early administration of the third-generation EGFR TKIs which are now used as the first line of treatment, some patients progress earlier than others. The development of resistance to EGFR TKIs has been thoroughly studied but, to date, no biomarker of response duration has been identified, except the presence of activating mutations in the *EGFR* gene itself. To address this gap, we conducted a study analyzing tumors from 135 lung cancer patients (including 56 patients with *EGFR*-mutant tumors and 79 with *EGFR*-WT tumors) using whole-exome sequencing.

We first confirmed that *KRAS* mutations were mutually exclusive with *EGFR* mutations. Moreover, we observed that three other genes were mutated only in *EGFR*-WT tumors: *KEAP1*, *STK11*, and *UNC80*. Two of these genes: *STK11* and *KEAP1*, are known to impact response to immunotherapy whereas the third one: *UNC80*, encodes a component of an ion channel complex and has never been found to be directly involved in any cancer-related mechanism [1]. However, the UNC80 protein has been shown to bind Src kinases [2] that do play some role in cancer progression. We did not identify any recurrent somatic variations associated with *EGFR* mutations. Nonetheless, we observed a significant enrichment of four SNPs: *CHRNA3* rs66793222*, COL18A1 c.3378_3386del, PLBD1* rs147342083, and *ZFPM1* rs149145771*,* in *EGFR*-mutant tumors.

The *CHRNA3* gene codes for a member of the nicotinic acetylcholine receptor family. Polymorphisms in this gene have been reported to be associated with heavy smoking [3], but also with susceptibility to lung cancer in the Chinese population [4]. We hypothesize that the enrichment of the *CHRNA3* rs66793222 variant we observed in patients with *EGFR*-mutant tumors compared to the reference Non-Finnish European (NFE) population may reflect a possible preventive effect of this SNP on tobacco dependence (people with this SNP are less susceptible to develop nicotine dependence). Indeed, it is well known that *EGFR* mutations are more frequent in lung tumors in never-smokers, and the lower frequency of *rs66793222* in *EGFR*-WT tumors seems to support this hypothesis.

The second SNP we identified was an in-frame deletion c.3378_3386del in the *COL18A1* gene that encodes the alpha chain of type XVIII collagen. Its frequency in patients with *EGFR*-WT tumors was similar to that in the NFE population. On the contrary, the frequency of this SNP in *EGFR*-mutant tumors was 12-fold higher than in NFE population, with more than 32% of patients with *EGFR*-mutant tumors harboring this SNP. While this particular SNP has not been described in the literature, another SNP in the *COL18A1* gene, *D104N*, has been shown to increase the risk of developing osteosarcoma [5] and sporadic breast cancer [6]. Our results suggest that the c.3378_3386del polymorphism might be associated with an increased prevalence of *EGFR*-mutant lung cancer. However, further studies are needed to confirm this hypothesis.

The two other SNPs enriched in patients with *EGFR*-mutant tumors were *PLBD1* rs147342083 and *ZFPM1* rs149145771. *PLBD1* codes for a phospholipase expressed in neutrophils, with its exact role being poorly understood. On the other hand, whereas the *ZFPM1* gene encodes a transcription factor, FOG1, well known for its role in erythroid and megakaryocytic cell differentiation [7]. We found that the frequencies of both variants in these two genes were very low in *EGFR*-WT tumors compared to the NFE population, suggesting that these SNPs might protect from *EGFR*-WT lung cancer.

While the presence of an *EGFR* mutation in the tumor is a known biomarker of sensitivity to EGFR TKIs, no other marker predicting the duration of response to EGFR TKI treatment has ever been reported in the literature. Therefore, we investigated whether the presence of variants in some other genes found using the exome analysis might influence progression-free survival (PFS) in patients receiving EGFR TKI. We found that polymorphisms in four genes—*CTBP2, HLA-DRB5*, *KIR3DL1*, and *KIR3DL2*—might have an impact. The *CTBP2* gene codes for a transcriptional repressor involved in tumorigenesis and cancer progression. Moreover, a high mRNA expression of CTBP2 was linked to a poor prognosis in patients with different cancers, including lung adenocarcinoma [8]. In our study, we focused on nucleotide variations in the *CTBP2* gene. It appeared that patients with the *CTBP2* reference genotype had a worse PFS than those harboring one SNP in this gene, and those with one SNP fared worse than those with two SNPs in the gene. This suggests that the *CTBP2* genotype may impact the activity of the resulting protein, particularly when it is overexpressed in cancer. Further analysis of the Wnt/β-catenin pathway activation, that could be triggered by CTBP2, may provide additional insights into the effect of the *CTBP2* genotype on patient survival.

On the contrary, the presence of a SNP in *HLA-DRB5*, *KIR3DL1*, and/or *KIR3DL2* immune-related genes decreased PFS of patients receiving EGFR TKI. The HLA-DRB5 mRNA expression has previously been associated with a better prognosis in lung adenocarcinoma [9]. In our study, the presence of any of the three aforementioned SNPs was significantly associated with worse PFS in patients receiving EGFR TKIs. This suggests that immunity may play a role in response to EGFR TKIs. This hypothesis is reinforced by our finding that particular *KIR3DL1* and *KIR3DL2* SNPs may impact the PFS of patients receiving such a treatment. Indeed, as for *HLA-DRB5*, patients with the reference sequence of *KIR3DL1* and *KIR3DL2* had a better PFS than those with at least one of the identified SNPs. Interestingly, we observed that patients with the reference sequence of *KIR3DL1* or *KIR3DL2* had also a better OS than those harboring at least one SNP in both genes. In addition, another gene of the KIR family, *KIR2DL4*, had a similar impact on survival as *KIR3DL1* and *KIR3DL2*, with better PFS for patients with the reference sequence than those who had at least one SNP in this gene. Of note, even though we detected these SNPs in the tumor exome analysis, they are also present in non-cancer cells, including immune cells. *KIR3DL1/2* and *KIR2DL4* are killer immunoglobulin-like receptors (KIR), mainly expressed by Natural Killer (NK) cells. They belong to haplotype A, present in about 50% of humans [10], and localized in the telomeric KIR region. It seems that ˗ at least in the South European population ˗ *KIR3DL2* and *KIR2DL4* are present in 100% of KIR profiles, and *KIR3DL1* is present in almost 82% of profiles [11]. KIRs interact with HLA class I molecules. When KIRs detect a self-molecule, NK cells are inhibited, avoiding the destruction of normal cells. When they detect a tumor cell, the interaction between HLA class I molecule with KIR activates NK cells to destroy abnormal cells. EGFR TKIs were shown to increase the expression of HLA class I molecules at the surface of tumor cells [12]. They also increase the tumor-induced chemotaxis of immune cells, which results in an increased tumor infiltration by immune cells, including NK cells [12]. In addition, it was reported that erlotinib treatment of tumors with the p.(Leu858Arg) *EGFR*-mutation enhances the antigen-presenting capabilities of antigen-presenting cells [13]. Finally, the hypothesis that the involvement of NK cells is important for effective treatment of *EGFR*-mutant lung adenocarcinomas is supported by the positive impact of autologous NK cell immunotherapy in advanced lung adenocarcinoma, especially in patients with *EGFR*-mutant tumors [14]. Of note, a phase I/II clinical trial studying the combination of natural killer T cells and gefitinib in the treatment of advanced *EGFR*-mutant lung cancer started is currently underway [15].

## Conclusions

In conclusion, our work shows that SNPs may impact the response to tumor-targeting therapeutics such as tyrosine kinase inhibitors in lung cancer patients, probably by affecting the tumor microenvironment and non-tumor cells, especially immune cells.

## Supplementary Information

**Additional file 1.** Supplementary Results.

**Additional file 2: Supplementary Figure S1. **Progression-free survival (PFS) rates for patients with *EGFR*-mutant and -wildtype (WT) lung cancer. Patients with *EGFR*-mutant tumors were stratified by (A) their mutation, and (B) their treatment. Then, patients were stratified according to (C) *EGFR* status of the tumor, (D) performance status, (E) metastatic sites, (F) sex, and (G) disease stage.

**Additional file 3: Supplementary Figure S2. **Progression-free survival (PFS) rates for patients with *EGFR*-mutant (A) and -wildtype (B) lung cancer by the disease stage at diagnosis.

**Additional file 4: Supplementary Figure S3.** Some germline polymorphisms are enriched in *EGFR*-mutant tumors (A) Heatmap of gene variants whose frequencies differed between *EGFR-WT *and *EGFR-mutant* (M+) tumors. (B) Barplots displaying the proportions of SNPs enriched in *EGFR-mutant* (M+) tumors compared to *EGFR*-WT tumors. NFE: non-Finnish European population.

**Additional file 5: Supplementary Table S1.** Patients and tumors - demographic and clinical characteristics.

**Additional file 6: Supplementary Table S2.** An overview of recurrent mutations detected in *EGFR*-wildtype (WT) lung tumors.

**Additional file 7: Supplementary Table S3.** The prevalence of germline polymorphisms (SNPs) significantly enriched in *EGFR*-mutant tumors compared to that in *EGFR*-wildtype (WT) tumors and to the reference non-Finnish European population (NFE, corresponds to Caucasian population).

**Additional file 8: Supplementary Table S4.** An overview of single nucleotide polymorphisms (SNPs) which correlated with the overall survival of patients with *EGFR*-mutant tumors treated with an EGFR tyrosine kinase inhibitor.

## Data Availability

Genomic data may be shared upon reasonable request to the corresponding author in accordance with the French law on genomic data.
