# Supplementary material for: Durable response of lung carcinoma patients to EGFR tyrosine kinase inhibitors is determined by germline polymorphisms in some immune-related genes
Source: Mol Cancer. 2023 Jul 29;22:120. doi: 10.1186/s12943-023-01829-4 (PMC10385908; doi:10.1186/s12943-023-01829-4)
Supplement: Supplementary file 1 — Additional file 1. Supplementary Results. [file 12943_2023_1829_MOESM1_ESM.docx]

**Supplementary information _ Results**

*Patients and tumors*

The basic demographic and clinical characteristics of patients and tumors are summarized in Supplementary Table S1.

**Supplementary Table S1. Patients and tumors - demographic and clinical characteristics.**

|  | **Total**  **(n = 135)** | | ***EGFR*-mutant tumors**  **(n = 56)** | | ***EGFR-wildtype* tumors**  **(n = 79)** | | ***p*** |
| --- | --- | --- | --- | --- | --- | --- | --- |
| **Feature** | **n** | **%** | **n** | **%** | **n** | **%** |  |
| **Patient age, (years)** |  |  |  |  |  |  | *0.001* |
| Mean | 64.2 |  | 67.9 |  | 61.6 |  |  |
| Standard deviation | 11.3 |  | 11.0 |  | 10.9 |  |  |
| **Sex** |  |  |  |  |  |  | *< 0.001* |
| Male | 76 | 56.0 | 18 | 32.1 | 58 | 73.4 |  |
| Female | 59 | 44.0 | 38 | 67.9 | 31 | 26.6 |  |
| **Disease stage** |  |  |  |  |  |  |  |
| I – IIIA | 9 | 6.7 | 5 | 8.9 | 4 | 5.1 |  |
| IIIB – IV | 126 | 93.3 | 51 | 91.1 | 75 | 94.9 |  |
| **Tumor histology** |  |  |  |  |  |  |  |
| Adenocarcinoma | 119 | 88.1 | 56 | 100 | 63 | 79.8 |  |
| Squamous cell carcinoma | 12 | 8.9 |  |  | 12 | 15.2 |  |
| Large cell neuroendocrine carcinoma | 2 | 1.5 |  |  | 2 | 2.5 |  |
| Large cell carcinoma | 1 | 0.75 |  |  | 1 | 1.25 |  |
| NA | 1 | 0.75 |  |  | 1 | 1.25 |  |
| **Smoking status** |  |  |  |  |  |  |  |
| Ever smoker | 39 | 28.9 | 11 | 19.6 | 28 | 35.4 |  |
| Never smoker | 17 | 12.6 | 9 | 16.1 | 8 | 10.1 |  |
| NA | 79 | 58.5 | 36 | 64.3 | 43 | 54.5 |  |
| **Metastatic site** |  |  |  |  |  |  |  |
| Brain and/or liver | 60 | 44.4 | 25 | 44.6 | 35 | 44.3 |  |
| Other site | 63 | 46.7 | 20 | 35.7 | 43 | 54.4 |  |
| NA | 12 | 8.9 | 11 | 19.7 | 1 | 1.3 |  |
| **Performance status** |  |  |  |  |  |  |  |
| 0-1 | 118 | 87.4 | 51 | 91.1 | 67 | 84.8 |  |
| 2-3 | 16 | 11.9 | 5 | 8.9 | 11 | 13.9 |  |
| NA | 1 | 0.7 |  |  | 1 | 1.3 |  |
| ***EGFR* status** |  |  |  |  |  |  |  |
| Wild-type | 79 | 59.0 |  |  | 79 | 100 |  |
| p.(Gly719X) | 4 | 3.0 | 4 | 7.1 |  |  |  |
| Exon-19 del | 29 | 21.5 | 29 | 51.8 |  |  |  |
| Exon-20 mutations | 2 | 1.5 | 2 | 3.6 |  |  |  |
| p.(Leu858Arg) | 21 | 16.0 | 21 | 37.5 |  |  |  |
| **EGFR-TKI treatment** |  |  |  |  |  |  |  |
| Afatinib |  |  | 47 | 83.9 |  |  |  |
| Gefitinib |  |  | 4 | 7.1 |  |  |  |
| Afatinib then gefitinib |  |  | 4 | 7.1 |  |  |  |
| Afatinib then erlotinib |  |  | 1 | 1.8 |  |  |  |
| **Response to 1^st^ line treatment** | |  |  |  |  |  | *0.01* |
| PD | 39 | 28.9 | 9 | 16.1 | 30 | 38.0 |  |
| SD | 21 | 15.6 | 7 | 12.5 | 14 | 17.7 |  |
| PR | 55 | 40.7 | 31 | 55.4 | 24 | 30.4 |  |
| CR | 10 | 7.4 | 4 | 7.1 | 6 | 7.6 |  |
| NA | 10 | 7.4 | 5 | 8.9 | 5 | 6.3 |  |

Abbreviations

CR: complete response; EGFR: Epidermal Growth Factor Receptor; NA: not available; PD: progressive disease; PR: partial response; SD: stable disease; STDEV: standard deviation; TKI: tyrosine kinase inhibitor

Of the total 165 patients with lung cancer who were enrolled in the ALCAPONE trial, 135 had enough tumor material for genomic analysis (HES > 20%). Fifty-six of them (41.5%) had *EGFR*-mutant tumors, while 79 (58.5%) had tumors with wild-type *EGFR*. *EGFR*-mutant tumors were enriched compared to classical figures due to a specific selection of patients with *EGFR*-mutant tumors during the second half of the trial. This was decided to reach a number of *EGFR*-mutant samples that would be sufficient to obtain statistical significance in the analyses. The mean age of the study population was 64.2 years, with a significant age difference between patients with *EGFR*-mutant tumors and those with *EGFR*-WT tumors (67.9 versus 61.6 years; *p* = 0.001). Also male-to-female ratios significantly differed between the two groups, with a higher proportion of women among patients with *EGFR*-mutant tumors (*p* < 0.001). A majority of patients (125; 93.3%) had a stage IIIB or IV disease. Patients with an earlier-stage disease were treated with EGFR TKI or chemotherapy, according to the *EGFR* status of their tumors, after stage IV relapse. Both progression-free and overall survival were calculated from the date at which the metastatic disease was diagnosed.

*Tumor EGFR status affects response to treatment and patient survival*

The most prevalent *EGFR* gene variation in *EGFR*-mutant tumors was an in-frame deletion in exon 19, which was detected in half of the patients (n=29), followed by the p.(Leu858Arg) mutation (n=21), two p.(Gly719X) mutations: (p.(Gly719Arg)*3 and p.(Gly719Ser)*1), and finally mutations in exon 20 (in-frame insertion *1 and p.(Ser768Ile)+p.(Val769Leu)*1). Except for mutations in exon 20, which were associated with no response to treatment, other activating mutations did not appear to affect progression-free survival of the patients (Supplementary Figure S1A).

The majority of patients with *EGFR*-mutant tumors were treated with afatinib (n=47). Five other patients started with afatinib but quickly switched to gefitinib (n=4) or erlotinib (n=1) due to afatinib toxicity. Finally, four patients were treated only with gefitinib. We did not observe any differences in PFS between the different treatment schemes (Supplementary Figure S1B). In the whole study population, 39 (28.9%) patients did not respond to treatment at all. The response rates significantly differed between patients with *EGFR*-mutant and those with *EGFR*-WT tumors (*p* = 0.01; Supplementary Table S1). Patients treated with an EGFR TKI (all those with *EGFR*-mutant tumors) had higher response rates than those treated with chemotherapy (patients with *EGFR*-WT tumors; 71.9% versus 55.7%). This was also reflected by a significant difference in the progression-free survival (PFS) between the two groups (Supplementary Figure S1C).


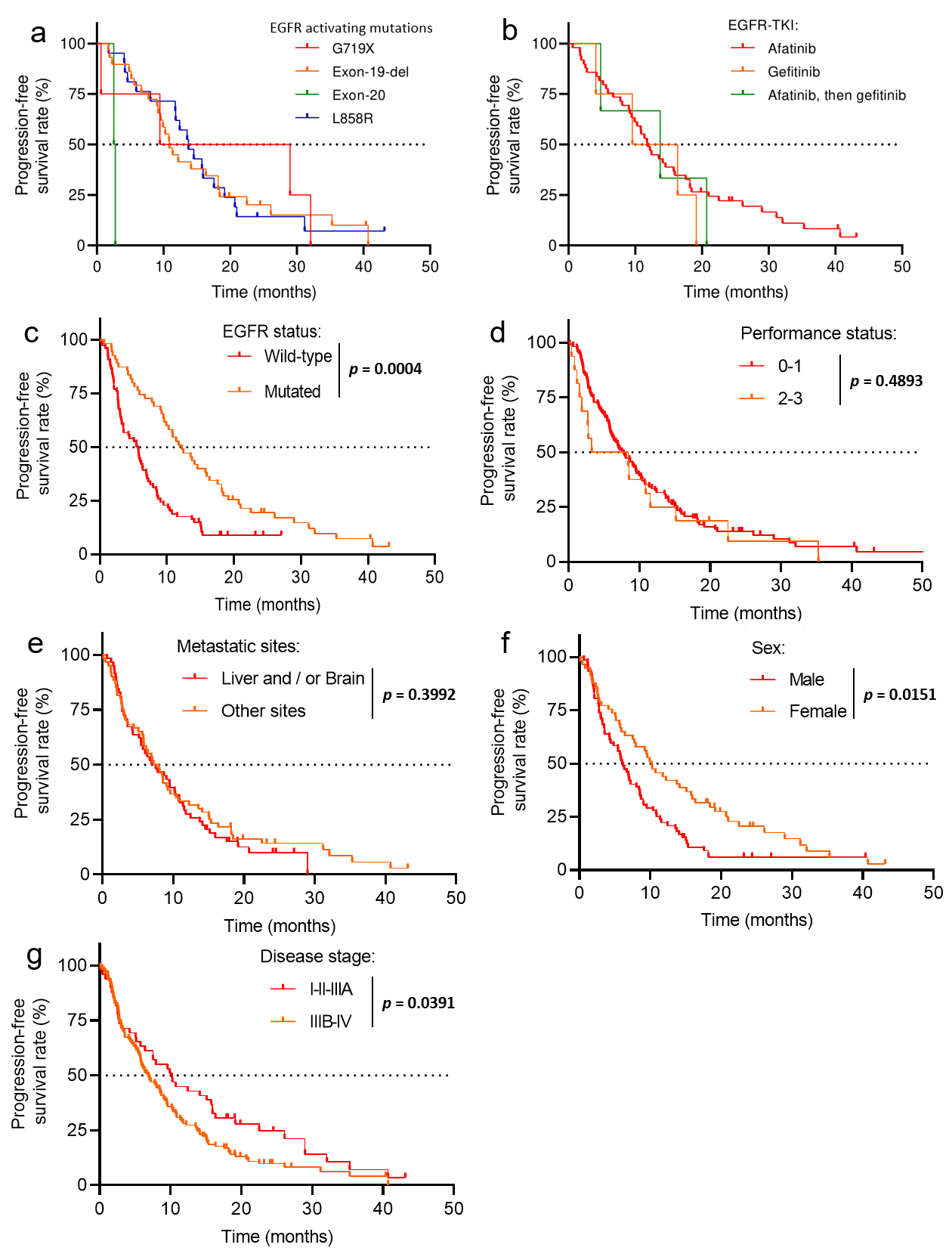


**Supplementary Figure S1.** Progression-free survival (PFS) rates for patients with *EGFR*-mutant and -wildtype (WT) lung cancer. Patients with *EGFR*-mutant tumors were stratified by **(A)** their mutation, and **(B)** their treatment. Then, patients were stratified according to **(C)** *EGFR* status of the tumor, **(D)** performance status, **(E)** metastatic sites, **(F)** sex, and **(G)** disease stage.

Neither performance status (Supplementary Figure S1D), nor metastatic sites (Supplementary Figure S1E) appeared to affect patient response to treatment and/or survival. It seemed that sex of patients and disease stage might impact PFS (Supplementary Figure S1F-G), with a higher survival rate for women and for patients with stage I to IIIA disease, however, these results did not reach the threshold of statistical significance. Of note, there was a higher proportion of women among patients with *EGFR*-mutant tumors and more than a half of stage I - IIIA disease patients belonged to the *EGFR*-mutant subset. Nevertheless, when analyzing the impact of disease stage on progression-free survival separately for patients with *EGFR*-mutant tumors and for those with *EGFR*-WT tumors, we did not observe any significant difference between the two groups (Supplementary Figure S2).


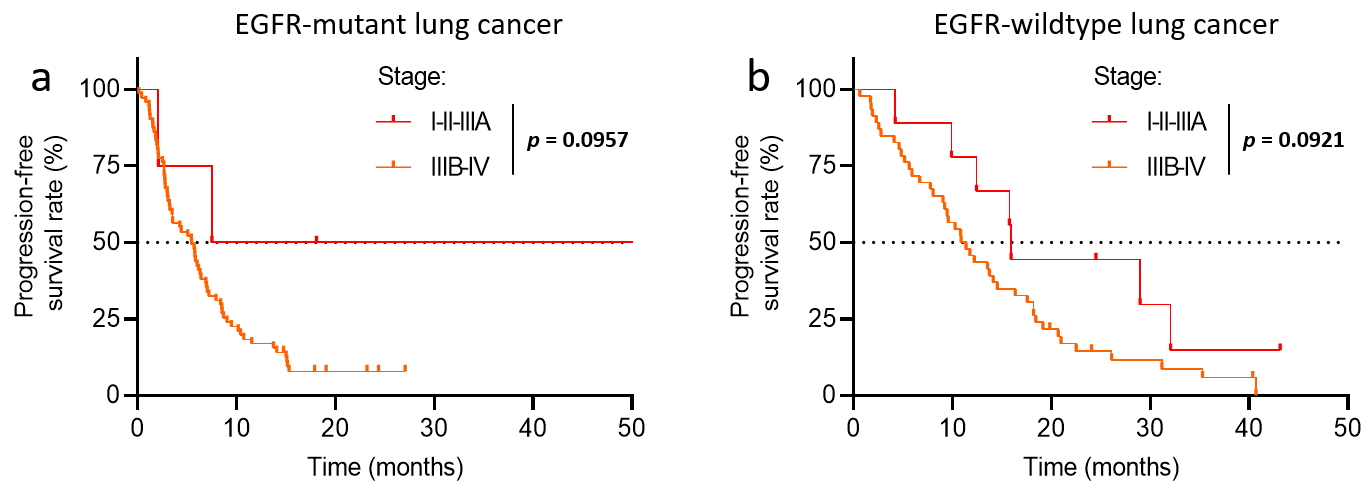


**Supplementary Figure S2.** Progression-free survival (PFS) rates for patients with *EGFR*-mutant (**A**) and -wildtype (**B**) lung cancer by the disease stage at diagnosis.
