# Supplementary figures and images for: Durable response of lung carcinoma patients to EGFR tyrosine kinase inhibitors is determined by germline polymorphisms in some immune-related genes
Source: Mol Cancer. 2023 Jul 29;22:120. doi: 10.1186/s12943-023-01829-4 (PMC10385908; doi:10.1186/s12943-023-01829-4)

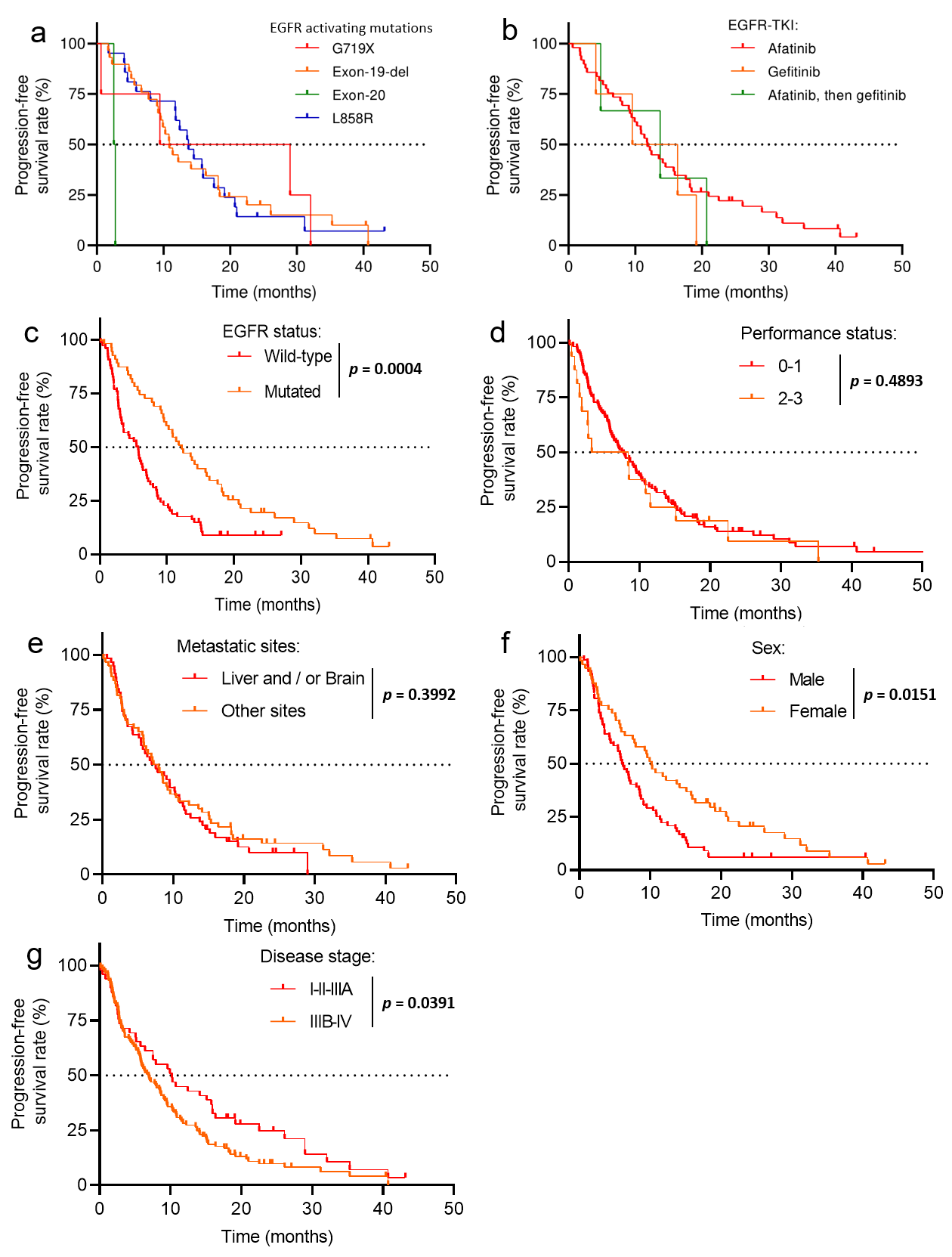

Supplement: Supplementary file 2 — Additional file 2: Supplementary Figure S1. Progression-free survival (PFS) rates for patients with EGFR-mutant and -wildtype (WT) lung cancer. Patients with EGFR-mutant tumors were stratified by (A) their mutation, and (B) their treatment. Then, patients were stratified according to (C) EGFR status of the tumor, (D) performance status, (E) metastatic sites, (F) sex, and (G) disease stage. [file 12943_2023_1829_MOESM2_ESM.tif]

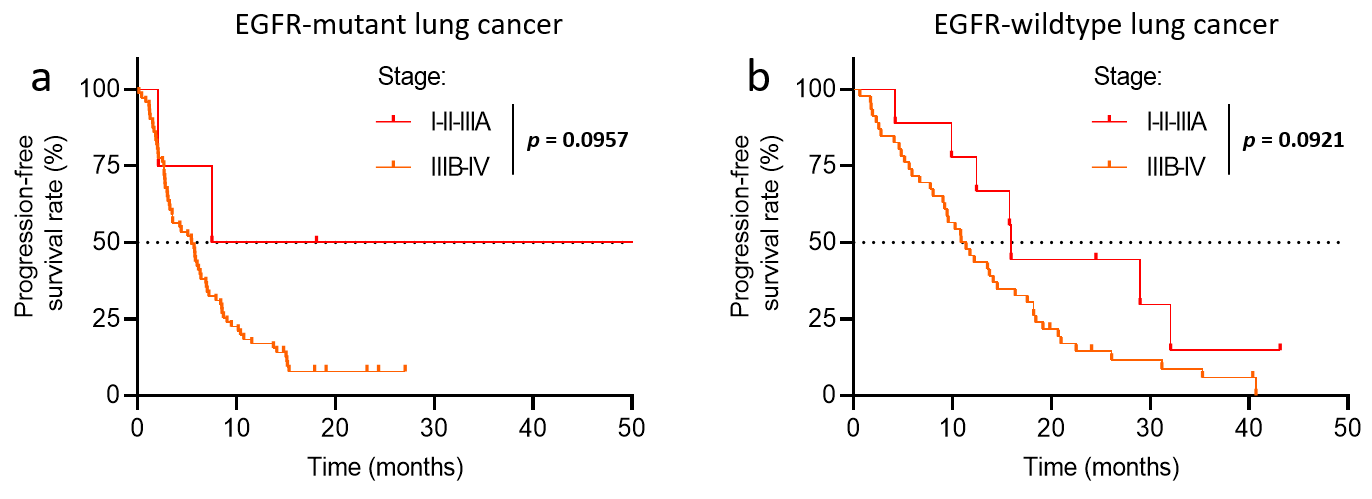

Supplement: Supplementary file 3 — Additional file 3: Supplementary Figure S2. Progression-free survival (PFS) rates for patients with EGFR-mutant (A) and -wildtype (B) lung cancer by the disease stage at diagnosis. [file 12943_2023_1829_MOESM3_ESM.tif]

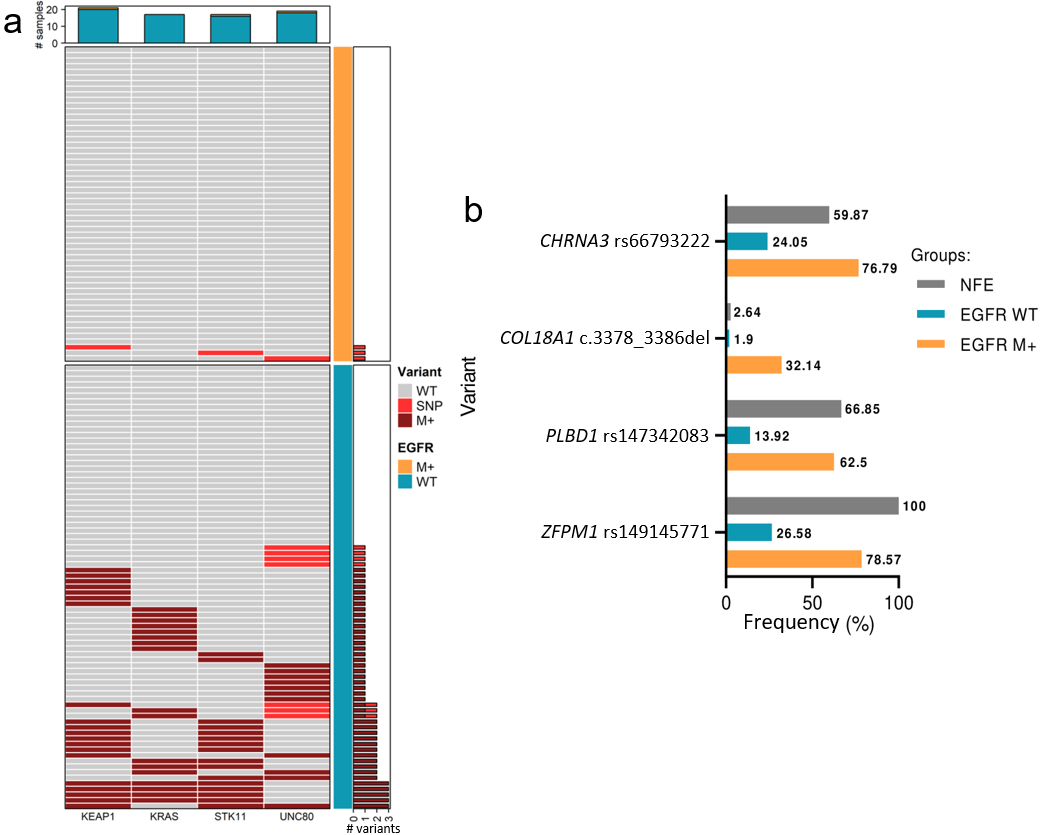

Supplement: Supplementary file 4 — Additional file 4: Supplementary Figure S3. Some germline polymorphisms are enriched in EGFR-mutant tumors (A) Heatmap of gene variants whose frequencies differed between EGFR-WT and EGFR-mutant (M+) tumors. (B) Barplots displaying the proportions of SNPs enriched in EGFR-mutant (M+) tumors compared to EGFR-WT tumors. NFE: non-Finnish European population. [file 12943_2023_1829_MOESM4_ESM.tif]
