# Supplementary material for: Durable response of lung carcinoma patients to EGFR tyrosine kinase inhibitors is determined by germline polymorphisms in some immune-related genes
Source: Mol Cancer. 2023 Jul 29;22:120. doi: 10.1186/s12943-023-01829-4 (PMC10385908; doi:10.1186/s12943-023-01829-4)
Supplement: Supplementary file 5 — Additional file 5: Supplementary Table S1. Patients and tumors - demographic and clinical characteristics. [file 12943_2023_1829_MOESM5_ESM.doc]

**Supplementary Table S1. Patients and tumors - demographic and clinical characteristics.**

|  | **Total**  **(n = 135)** | | ***EGFR*-mutant tumors**  **(n = 56)** | | ***EGFR-wildtype* tumors**  **(n = 79)** | | ***p*** |
| --- | --- | --- | --- | --- | --- | --- | --- |
| **Feature** | **n** | **%** | **n** | **%** | **n** | **%** |
| **Patient age, (years)** |  |  |  |  |  |  | *0.001* |
| Mean | 64.2 |  | 67.9 |  | 61.6 |  |  |
| Standard deviation | 11.3 |  | 11.0 |  | 10.9 |  |  |
| **Sex** |  |  |  |  |  |  | *< 0.001* |
| Male | 76 | 56.0 | 18 | 32.1 | 58 | 73.4 |  |
| Female | 59 | 44.0 | 38 | 67.9 | 31 | 26.6 |  |
| **Disease stage** |  |  |  |  |  |  |  |
| I – IIIA | 9 | 6.7 | 5 | 8.9 | 4 | 5.1 |  |
| IIIB – IV | 126 | 93.3 | 51 | 91.1 | 75 | 94.9 |  |
| **Tumor histology** |  |  |  |  |  |  |  |
| Adenocarcinoma | 119 | 88.1 | 56 | 100 | 63 | 79.8 |  |
| Squamous cell carcinoma | 12 | 8.9 |  |  | 12 | 15.2 |  |
| Large cell neuroendocrine carcinoma | 2 | 1.5 |  |  | 2 | 2.5 |  |
| Large cell carcinoma | 1 | 0.75 |  |  | 1 | 1.25 |  |
| NA | 1 | 0.75 |  |  | 1 | 1.25 |  |
| **Smoking status** |  |  |  |  |  |  |  |
| Ever smoker | 39 | 28.9 | 11 | 19.6 | 28 | 35.4 |  |
| Never smoker | 17 | 12.6 | 9 | 16.1 | 8 | 10.1 |  |
| NA | 79 | 58.5 | 36 | 64.3 | 43 | 54.5 |  |
| **Metastatic site** |  |  |  |  |  |  |  |
| Brain and/or liver | 60 | 44.4 | 25 | 44.6 | 35 | 44.3 |  |
| Other site | 63 | 46.7 | 20 | 35.7 | 43 | 54.4 |  |
| NA | 12 | 8.9 | 11 | 19.7 | 1 | 1.3 |  |
| **Performance status** |  |  |  |  |  |  |  |
| 0-1 | 118 | 87.4 | 51 | 91.1 | 67 | 84.8 |  |
| 2-3 | 16 | 11.9 | 5 | 8.9 | 11 | 13.9 |  |
| NA | 1 | 0.7 |  |  | 1 | 1.3 |  |
| ***EGFR* status** |  |  |  |  |  |  |  |
| Wild-type | 79 | 59.0 |  |  | 79 | 100 |  |
| p.(Gly719X) | 4 | 3.0 | 4 | 7.1 |  |  |  |
| Exon-19 del | 29 | 21.5 | 29 | 51.8 |  |  |  |
| Exon-20 mutations | 2 | 1.5 | 2 | 3.6 |  |  |  |
| p.(Leu858Arg) | 21 | 16.0 | 21 | 37.5 |  |  |  |
| **EGFR-TKI treatment** |  |  |  |  |  |  |  |
| Afatinib |  |  | 47 | 83.9 |  |  |  |
| Gefitinib |  |  | 4 | 7.1 |  |  |  |
| Afatinib then gefitinib |  |  | 4 | 7.1 |  |  |  |
| Afatinib then erlotinib |  |  | 1 | 1.8 |  |  |  |
| **Response to 1^st^ line treatment** | |  |  |  |  |  | *0.01* |
| PD | 39 | 28.9 | 9 | 16.1 | 30 | 38.0 |  |
| SD | 21 | 15.6 | 7 | 12.5 | 14 | 17.7 |  |
| PR | 55 | 40.7 | 31 | 55.4 | 24 | 30.4 |  |
| CR | 10 | 7.4 | 4 | 7.1 | 6 | 7.6 |  |
| NA | 10 | 7.4 | 5 | 8.9 | 5 | 6.3 |  |

Abbreviations

CR: complete response; EGFR: Epidermal Growth Factor Receptor; NA: not available; PD: progressive disease; PR: partial response; SD: stable disease; STDEV: standard deviation; TKI: tyrosine kinase inhibitor
