# Supplementary material for: Durable response of lung carcinoma patients to EGFR tyrosine kinase inhibitors is determined by germline polymorphisms in some immune-related genes
Source: Mol Cancer. 2023 Jul 29;22:120. doi: 10.1186/s12943-023-01829-4 (PMC10385908; doi:10.1186/s12943-023-01829-4)
Supplement: Supplementary file 6 — Additional file 6: Supplementary Table S2. An overview of recurrent mutations detected in EGFR-wildtype (WT) lung tumors. [file 12943_2023_1829_MOESM6_ESM.doc]

Supplementary Table S2. An overview of recurrent mutations detected in *EGFR*-wildtype (WT) lung tumors.

| Gene | Nucleotide variant | Protein variant | Impact | Group |
| --- | --- | --- | --- | --- |
| *KEAP1* | c.99C>G | p.(Tyr33*) | LoF | WT *EGFR* |
|  | c.1174G>T | p.(Ala392Ser) | Unknown | WT *EGFR* |
|  | c.1249G>T | p.(Gly417Trp) | LoF | WT *EGFR* |
|  | c.1439G>T | p.(Gly480Val) | LoF | WT *EGFR* |
|  | c.1532-1G>T | *splicing* | LoF | WT *EGFR* |
|  | c.1603G>T | p.(Glu535*) | LoF | WT *EGFR* |
|  | c.1715A>G | p.(Tyr572Cys) | LoF | WT *EGFR* |
|  | c.1801C>T | p.(Arg601Trp) | LoF | WT *EGFR* |
|  | c.283G>T | p.(Ala95Ser) | Unknown | WT *EGFR* |
|  | c.482T>C | p.(Met161Thr) | Unknown | WT *EGFR* |
|  | c.483G>A | p.(Met161Ile) | LoF | WT *EGFR* |
|  | c.483G>T | p.(Met161Ile) | LoF | WT *EGFR* |
|  | c.512G>T | p.(Cys171Phe) | Unknown | WT *EGFR* |
|  | c.557G>A | p.(Gly186Asp) | Unknown | WT *EGFR* |
|  | c.574G>C | p.(Glu192Gln) | SNP | Mutant *EGFR* |
|  | c.652G>C | p.(Glu218Gln) | LoF | WT *EGFR* |
|  | c.730G>A | p.(Glu244Lys) | LoF | WT *EGFR* |
|  | c.736T>G | p.(Phe246Val) | Unknown | WT *EGFR* |
|  | c.840C>A | p.(Phe280Leu) | Unknown | WT *EGFR* |
|  | c.852G>T | p.(Gln284His) | Unknown | WT *EGFR* |
|  | c.896A>G | p.(Asp299Gly) | Unknown | WT *EGFR* |
| *KRAS* | c.34G>T | p.(Gly12Cys) | GoF | WT *EGFR* (n=9) |
|  | c.35G>C | p.(Gly12Arg) | GoF | WT *EGFR* (n=2) |
|  | c.35G>T | p.(Gly12Val) | GoF | WT *EGFR* (n=5) |
|  | c.37G>T | p.(Gly13Cys) | GoF | WT *EGFR* |
| *STK11* | c.109C>T | p.(Gln37*) | LoF | WT *EGFR* |
|  | c.1211C>T | p.(Ser404Phe) | SNP | Mutant *EGFR* |
|  | c.250A>T | p.(Lys84*) | LoF | WT *EGFR* |
|  | c.291_292insT | p.(Glu98*) | LoF | WT *EGFR* |
|  | c.388G>T | p.(Glu130*) | LoF | WT *EGFR* |
|  | c.394dupT | p.(Cys132Leufs*31) | LoF | WT *EGFR* |
|  | c.402_403delTG | p.(Cys134Trpfs*28) | LoF | WT *EGFR* |
|  | c.465-1G>T | *splicing* | LoF | WT *EGFR* |
|  | c.597+2T>A | *splicing* | LoF | WT *EGFR* |
|  | c.598-2A>T | *splicing* | LoF | WT *EGFR* |
|  | c.752delG | p.(Gly251Valfs*36) | LoF | WT *EGFR* |
|  | c.766G>T | p.(Glu256*) | LoF | WT *EGFR* |
|  | c.785delA | p.(Lys262Serfs*25) | LoF | WT *EGFR* |
|  | c.836delG | p.(Gly279Alafs*8) | LoF | WT *EGFR* |
|  | c.897delC | p.(Ile300Serfs*36) | LoF | WT *EGFR* |
|  | c.920+1G>T | *splicing* | LoF | WT *EGFR* |
|  | c.991_992delCG | p.(Arg331Valfs*28) | LoF | WT *EGFR* |
| *UNC80* | c.95C>A | p.Ala32Glu | Unknown | WT *EGFR* |
|  | c.404C>T | p.Thr135Ile | SNP | WT *EGFR* (n=2) |
|  | c.1432C>A | p.Leu478Ile | Unknown | WT *EGFR* |
|  | c.1822A>G | p.Met608Val | SNP | WT *EGFR* |
|  | c.3074G>C | p.Arg1025Pro | Unknown | WT *EGFR* |
|  | c.3356G>C | p.Ser1119Thr | SNP | WT *EGFR* |
|  | c.3379G>T | p.Ala1127Ser | Unknown | WT *EGFR* |
|  | c.3434A>C | p.Glu1145Ala | SNP | WT *EGFR* |
|  | c.3475G>C | p.Asp1159His | Unknown | WT *EGFR* |
|  | c.3932C>T | p.Thr1311Ile | SNP | WT *EGFR* (n=2)  Mutant *EGFR* (n=1) |
|  | c.5048C>T | p.Ser1683Leu | Unknown | WT *EGFR* |
|  | c.6032A>T | p.Gln2011Leu | Unknown | WT *EGFR* |
|  | c.6605A>G | p.Glu2202Gly | Unknown | WT *EGFR* |
|  | c.7334C>T | p.Ser2445Leu | SNP | WT *EGFR* |
|  | c.8263A>G | p.Ser2755Gly | SNP | WT *EGFR* |
|  | c.8303C>A | p.Pro2768Gln | Unknown | WT *EGFR* |
|  | c.8330C>A | p.Ala2777Glu | Unknown | WT *EGFR* |
|  | c.9449C>G | p.Pro3150Arg | Unknown | WT *EGFR* |
|  | c.9482C>T | p.Ala3161Val | SNP | WT *EGFR* |

Abbreviations.

GoF: Gain-of-Function; LoF: Loss-of-Function; SNP: Single Nucleotide Polymorphism
