# Supplementary material for: Durable response of lung carcinoma patients to EGFR tyrosine kinase inhibitors is determined by germline polymorphisms in some immune-related genes
Source: Mol Cancer. 2023 Jul 29;22:120. doi: 10.1186/s12943-023-01829-4 (PMC10385908; doi:10.1186/s12943-023-01829-4)
Supplement: Supplementary file 7 — Additional file 7: Supplementary Table S3. The prevalence of germline polymorphisms (SNPs) significantly enriched in EGFR-mutant tumors compared to that in EGFR-wildtype (WT) tumors and to the reference non-Finnish European population (NFE, corresponds to Caucasian population). [file 12943_2023_1829_MOESM7_ESM.doc]

Supplementary Table S3. The prevalence of germline polymorphisms (SNPs) significantly enriched in *EGFR*-mutant tumors compared to that in *EGFR*-wildtype (WT) tumors and to the reference non-Finnish European population (NFE, corresponds to Caucasian population)

| SNP | Gene | Nucleotide variant | Protein variant | | NFE frequency | WT EGFR Variant frequency in *EGFR*-WT tumors | Variant frequency in *EGFR*-mutant tumors |
| --- | --- | --- | --- | --- | --- | --- | --- |
| rs66793222 | *CHRNA3* | c.67_69del | p.(Leu23del) | | 59.87% | 24.05% | 76.79% |
| NA | *COL18A1* | c.3378_3386del | p.(Pro1127_Pro1129del) | | 2.64% | 1.90% | 32.14% |
| rs147342083 | *PLBD1* | c.74_76del | | p.(Leu25del) | 66.85% | 13.92% | 62.50% |
| rs149145771 | *ZFPM1* | c.1335_1340del | p.(Leu446_Ala447del) | | 100% | 26.58% | 78.57% |

SNP: single nucleotide polymorphism
