# Supplementary material for: Durable response of lung carcinoma patients to EGFR tyrosine kinase inhibitors is determined by germline polymorphisms in some immune-related genes
Source: Mol Cancer. 2023 Jul 29;22:120. doi: 10.1186/s12943-023-01829-4 (PMC10385908; doi:10.1186/s12943-023-01829-4)
Supplement: Supplementary file 8 — Additional file 8: Supplementary Table S4. An overview of single nucleotide polymorphisms (SNPs) which correlated with the overall survival of patients with EGFR-mutant tumors treated with an EGFR tyrosine kinase inhibitor. [file 12943_2023_1829_MOESM8_ESM.doc]

Supplementary Table S4. An overview of single nucleotide polymorphisms (SNPs) which correlated with the overall survival of patients with *EGFR*-mutant tumors treated with an EGFR tyrosine kinase inhibitor.

| SNP | Gene | Nucleotide nomenclature | Protein nomenclature | HR [95% CI] | *p*-value |
| --- | --- | --- | --- | --- | --- |
| rs778020788 | *KIR3DL1* | c.1318G>A | p.(Val440Ile) | 4.33 [1.81-10.39] | 0.0004 |
| rs200731425 | *PRSS3* | c.685A>G | p.(Lys229Glu) | 3.47 [1.40-8.58] | 0.0042 |
| rs113792624 | *CTDSP3* | c.603G>T | p.(Lys201Asn) | 3.37 [1.29-8.81] | 0.0087 |
| rs77644642 | *ZNF217* | c.78G>T | p.(Glu26Asp) | 3.31 [1.52-7.20] | 0.0014 |
| rs189435670 | *HLA-DRB5* | c.590G>C | p.(Gly197Ala) | 3.13 [1.26-7.78] | 0.0097 |
| rs150899882 | *ARSD* | c.713G>T | p.(Cys238Phe) | 3.11 [1.33-7.31] | 0.0060 |
| rs143238998 | *ARSD* | c.719T>G | p.(Phe240Cys) |
| rs1136760 | *HLA-DRB5* | c.125A>G | p.(Tyr42Cys) | 3.05 [1.31-7.11] | 0.0068 |
| rs150280230 | *NOTCH4* | c.36_47del | p.(Leu13_Leu16del) | 2.84 [1.27-6.37] | 0.0081 |
| rs540514355 | *KIR2DL4* | c.484G>A | p.(Glu162Lys) | 2.50 [1.22-5.11] | 0.0094 |
| rs746343340 | *KIR2DL4* | c.446A>G | p.(Gln149Arg) | 2.45 [1.23-4.89] | 0.0083 |

Abbreviations.

CI: confidence interval; HR: hazard ratio
